# Activity evaluation of some psychoactive drugs with the application of QSAR/QSPR modeling methods

**DOI:** 10.1007/s00044-018-2234-5

**Published:** 2018-08-10

**Authors:** Piotr Kawczak, Leszek Bober, Tomasz Bączek

**Affiliations:** 1 Department of Pharmaceutical Chemistry, Faculty of Pharmacy with Subfaculty of Laboratory Medicine, Medical University of Gdańsk, Gdańsk, 80-416 Poland; 2 POLPHARMA SA Pharmaceutical Works, Starogard, Gdański, 83-200 Poland; 3 Institute of Health Sciences, Division of Human Anatomy and Physiology, Pomeranian University of Słupsk, Słupsk, 76-200 Poland

**Keywords:** Psychoactive drugs, Molecular modeling, Structural analysis, Descriptors, QSAR; QSPR

## Abstract

A set of psychoactive drugs has been analyzed with the use of quantitative structure–activity/property relationships methods. The purpose of this study was to demonstrate both the common and differentiating characteristics of the above-mentioned chemical compounds, physicochemical as well as pharmacological based on the quantum chemical calculations and selected biological activity data and chromatographic retention parameters. During the study, the ab initio model of molecular modeling was performed and PCA, FA, and MLR as the types of chemometric approach. QSAR/QSPR models were proposed based on chosen statistically significant descriptors. The relationship between the structure and biological activity data was able to class and describe the psychoactive properties of the molecules studied. The applied chemometric approaches revealed the influential features of tested structures responsible for their pharmacological activity together with some additional physicochemical properties.

## Introduction

The term psychoactive/psychotropic drug is defined as the collective name of substances whose only or main action is the effect on mental activities. In general, they are agents that act on the central nervous system, but there are also psychotropic agents in medicines belonging to other pharmacological groups. For pharmacological purposes, the division into psycholeptic, psychoanaleptic, and psychodysleptic drugs (hallucinogenic and psychotomimetic) is introduced. For practical purposes, people speak more often about neuroleptics, antidepressants, and anxiolytics. The pharmacological classification is also applied to the chemical classification taking into account the structure of the molecules of the considered drugs (Hendriksen and Groenink 2015; Stahl 2013; Dean 2011; Spiegel 2003).

Psychotropic agents have often been the subject of many works in the field of structure—properties, such as the relationship between the enthalpy of complexation of charge-transfer (CT) complexes with chloranil and their biological activity by a series of neuroleptics (Saucin and van der Vorst 1972), relationships between lipophilicity indices determined by RP-HPLC methods (as well as constants dissociation) and biological activity (Unger and Chiang 1981), pharmacological classification based on retention data in HPLC systems (Nasal et al. 1997) or attempts to predict biological activity for tricyclic neuroleptics and antidepressants based on quantum-chemical calculations of energy values of boundary orbitals e.g. HOMO and LUMO (Cogordan et al. 1999).

The subject of this work was the analysis for data from ab initio calculations and the biological activity data as well as physicochemical parameters presented in the cited papers (Saucin and van der Vorst 1972; Unger and Chiang 1981; Davis and Brody 1966). In addition, chromatographic retention data (Nasal et al. 1997) were also available for some of the compounds considered, which were used as variables dependent on structural parameters.

The aim of this research was to demonstrate both common and differentiating features of the analyzed chemical compounds both in physicochemical and pharmacological terms and confirmed the chemometrics relationship between nonempirical parameters characterizing chemical structure and biological activity.

## Materials and methods

### Molecules

**Figure 1:**
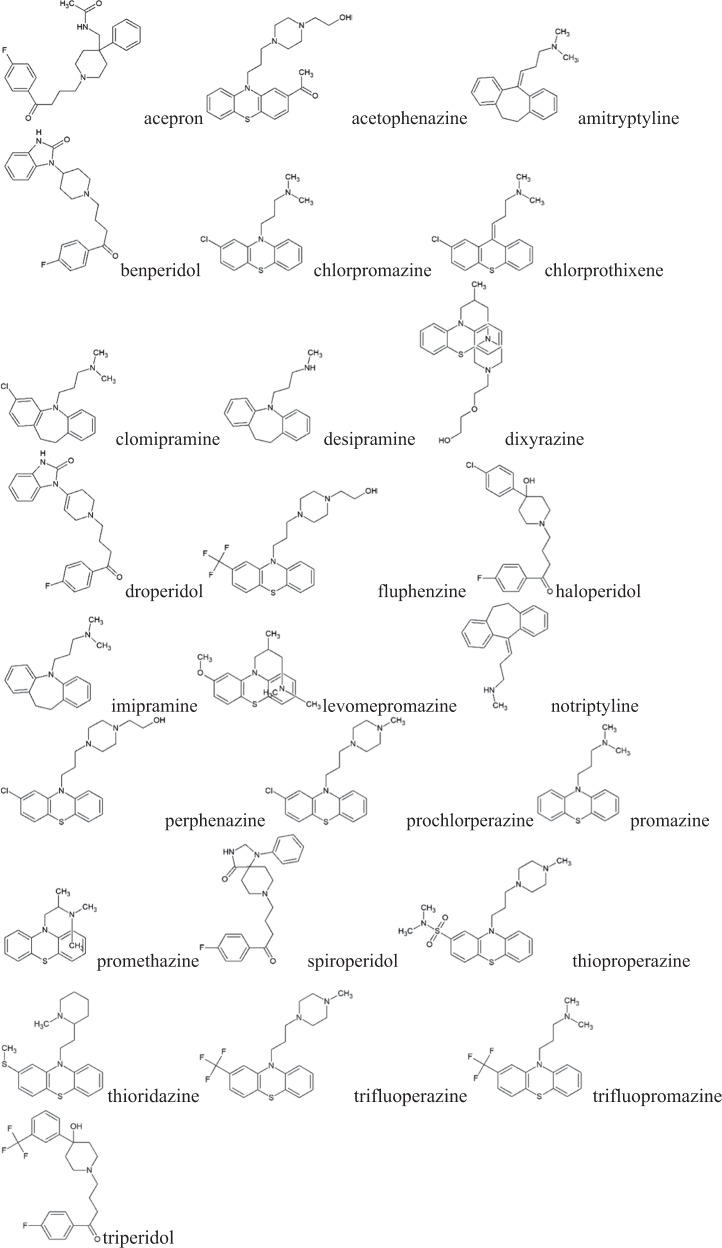
Structural formulas of compounds studied

The following compounds (Fig. 1), considered in the cited papers (Unger and Chiang 1981; Saucin and van der Vorst 1972; Davis and Brody 1966) were selected for the study: acoperon, benperidol, droperidol, haloperidol, spiroperidol, and triperidol from the group of butyrophenone neuroleptics, acetophenazine, chlorpromazine, chlorprothixen, dixyrazine, fluphenazine, levomepromazine, perphenazine, prochlorperazine, promazine, promethazine, thioproperazine, thioridazine, trifluoperazine, and trifluopromazine from the group of tricyclic neuroleptics; and: amitriptiline, clomipramine, desipramine, imipramine, and nortriptiline from the group of tricyclic antidepressants. Data on their structure were available for these compounds.

### Biological activity and physicochemical parameters data

The study includes literature data on biological activity: D dose (mg/kg) 50% reduction in motor activity of rats expressed as log (1/D) for neuroleptics of butyrophenone derivatives and parts of tricyclic neuroleptics (Saucin and van der Vorst 1972); histamine release as log (1/ED_50_ × 10^−3^) for parts of tricyclic neuroleptics and tricyclic antidepressants (Unger and Chiang 1981); ATPase activity inhibition as log [%/(100-%)] at 1 × 10^−4^ M for parts of tricyclic neuroleptics and imipramine (Davis and Brody 1966). The enthalpy values of −Δ*H* (kcal/mol) formation of neuroleptic CTs with chloranil (Saucin and van der Vorst 1972) obtained on the basis of spectroscopic measurements of CT complexes, and concern: neuroleptics of the butyrophenone and chlorpromazine derivatives, chlorprothixen, levomepromazine, prochlorperazine, and thioproperazines from the group of tricyclic neuroleptics.

### Chromatographic retention data

Chromatographic data comes from the work of Nasal et al. (1997) and relate to the compounds: chlorpromazine, chlorprothixene, fluphenazine, perphenazine, prochlorperazine, promazine, promethazine, thioridazine, trifluoperazine, trifluopromazine from the group of tricyclic neuroleptics, and: clomipramine, desipramine and imipramine from the group of tricyclic antidepressants. These are logarithm values of the retention coefficients determined on Chiral AGP (log *k*_AGP_) fillings and artificial IAM.PC.MG membranes (log *k*_IAM_) but also logarithm values of hydrophobicity coefficients determined by the polycratic method on suplex pKb-100 fillings pH 2.5 and 7.4 (log *k*_w2.5Su_, log *k*_w7.4Su_), Spheri RP-18 pH 2.5 and 7.0 (log *k*_w2.5Sp_, log *k*_w7.0Sp_), Aluspher RP select B pH 7.3 (log *k*_w7.3Al_) and Unisphere-PBD pH 11.7 (log *k*_w11.7Un_).

### Molecular descriptors

Nonempirical structural indicators—quantum-chemical indicators were calculated during the study. The structure of the tested compounds was studied by molecular modeling using the Gaussian 03W program (Gaussian Inc., Wallingford, CT, USA). The geometry of the molecules was optimized by the Hartree-Fock 6–31G (d, p) method (other designation is 6–31G**) (http://www.gaussian.com/). Among the quantum-chemical indicators were considered: total energy (TE), electronic spatial range (ESE)—the spatial extent of the molecule is defined as the surface covering the volume around the molecule beyond which the electron density is <0.001 eBohr^−3^, and describes the sensitivity of the molecule to the electric field), the energy of the highest occupied molecular orbitals (E_HOMO), the energy of the lowest unoccupied molecular orbitals (E_LUMO) and the energy difference HOMO and LUMO determined as energy gap (EG). In addition, the following values were used: the largest positive electron charge on atoms (MAX_POS), the largest negative electron charge on atoms (MAX_NEG), the difference between the highest positive and negative charge (Δ*Q*), total dipole moment (TDM), and isotropic polarization (IPOL). The values of total energy were expressed in atomic energy units a.u. or hartree (1 hartree = 2625.552 kJ·mol^−1^ or 627.5095 kcal·mol−1 or 27.2116 eV), HOMO energies, LUMO, and energy break were expressed in eV (the above values were converted from a.u. to eV), electronic spatial range in eBohr^−3^. The values of electron density and electron charges on atoms are in units of elementary charge (e^−^), dipole moment is expressed in debay (D) and isotropic polarizability in Bohr^3^ (Bohr = 0.5292·10^−10^ m = 0.5292 Å). Finally, for the whole group of molecules Dragon 7.0 (Kode Chemoinformatics, Pisa, Italy) software was used to calculate huge set – 5270 of extra descriptors (Todeschini and Consonni 2010; Dragon 7 molecular descriptors 2018 https://chm.kode-solutions.net/products_dragon.php).

### Statistical analysis

The data examined the biological activity, physicochemical and retention parameters of compounds were related to their structural indicators using multiparametric regression analysis/multiple regression analysis (MLR) with a stepwise progressive method together with principal component analysis (PCA) and factor analysis (FA) implemented in Statistica 13 (StatSoft, Tulsa, OK, USA) on a personal computer. In PCA, matrix of correlations has been diagonalized and in FA Varimax rotation has been performed.

## Results and discussion

The numerical values of all the 10 structural parameters derived from quantum-chemical calculations for all 25 compounds examined are shown in Table 1, the chromatographic retention parameters in Table 2 and finally the biological activity values and physicochemical parameters for selected compounds in Table 3.

**Table 1:** The numerical values of 10 structural parameters derived from Gaussian quantum-chemical calculations for all 25 compounds studied

Compound	TE	ESE	E_HOMO	E_LUMO	EG	MAX_POS	MAX_NEG	Δ*Q*	TDM	IPOL
Aceperon	−1283.84	21234.30	−8.9739	2.5415	11.5154	0.7254	−0.7283	1.4537	6.3837	242.90
Acetophenazine	−1598.45	14981.03	−7.6523	2.3717	10.0240	0.5402	−0.7950	1.3352	1.7719	259.79
Amitriptiline	−825.22	6515.35	−8.1413	3.5380	11.6793	0.1801	−0.5770	0.7571	1.1089	199.06
Benperidol	−1259.67	20740.83	−7.9418	2.6286	10.5704	1.0460	−0.8566	1.9026	3.1622	228.43
Chlorpromazine	−1620.72	8397.08	−7.7290	3.2460	10.9750	0.3143	−0.7846	1.0989	2.7007	201.34
Chlorprothixene	−1603.56	8999.30	−7.9483	2.6438	10.5921	0.2668	−0.5915	0.8583	2.2333	211.05
Clomipramine	−1301.28	8666.11	−8.0945	3.5056	11.6001	0.2790	−0.7897	1.0687	2.7070	204.08
Desipramine	−803.36	6526.89	−7.8879	3.7848	11.6727	0.2804	−0.7888	1.0692	0.9264	183.15
Dixyrazine	−1638.63	18634.72	−7.5078	3.5674	11.0752	0.3471	−0.8013	1.1484	1.4385	269.39
Droperidol	−1258.48	19155.09	−7.9960	2.5135	10.5095	1.0333	−0.8941	1.9274	5.3046	225.82
Fluphenazine	−1782.30	16638.00	−7.7804	2.9826	10.7630	1.1718	−0.7955	1.9673	4.7537	246.12
Haloperidol	−1571.75	17976.49	−8.7881	2.4400	11.2281	0.5509	−0.6697	1.2207	3.9820	220.16
Imipramine	−842.38	7331.05	−7.8795	3.7897	11.6692	0.2800	−0.7892	1.0693	0.7071	193.13
Levomepromazine	−1314.75	8340.75	−7.3870	3.6136	11.0006	0.4185	−0.8025	1.2209	1.5158	215.72
Nortriptiline	−786.19	6148.49	−8.0906	3.5102	11.6008	0.2710	−0.6326	0.9036	1.6374	189.70
Perphenazine	−1905.57	14014.45	−7.6751	3.2868	10.9619	0.3607	−0.7944	1.1551	3.8012	246.35
Prochlorperazine	−1791.68	11122.23	−7.6588	3.2955	10.9543	0.3613	−0.7944	1.1557	2.0514	233.31
Promazine	−1161.82	7343.41	−7.1788	3.5344	10.7132	0.3786	−0.8293	1.2078	2.5886	193.37
Promethazine	−1161.82	5419.31	−7.2528	3.6487	10.9015	0.3699	−0.8095	1.1793	2.1065	187.65
Spiroperidol	−1298.66	20148.70	−8.6112	2.6278	11.2390	0.7960	−0.7296	1.5256	4.3300	234.58
Thioproperazine	−2013.05	14948.37	−7.8264	2.6357	10.4621	1.7000	−0.7947	2.4947	5.9427	270.47
Thioridazine	−1714.32	10820.72	−7.6270	3.0993	10.7263	0.3470	−0.7869	1.1339	2.6694	247.11
Trifluoperazine	−1668.40	12802.53	−7.7701	2.9804	10.7505	1.1715	−0.7956	1.9672	2.5362	232.44
Trifluopromazine	−1497.44	10070.78	−7.8596	2.9567	10.8163	1.1740	−0.7842	1.9583	3.3624	200.41
Triperidol	−1448.48	21739.60	−8.8887	2.4065	11.2952	1.1740	−0.6691	1.8432	4.7615	219.02

**Table 2:** Chromatographic parameter values of selected antipsychoctic drugs

Compound	log k_AGP_	log k_IAM_	log k_w2.5Su_	log k_w7.4Su_	log k_w2.5Sp_	log k_w7.0Sp_	log k_w7.3Al_	log k_w11.7Un_
Chlorpromazine	2.131	1.435	1.595	4.051	1.935	2.632	3.309	4.076
Chlorprothixene	2.206	1.533	1.597	4.642	2.244	2.417	4.440	4.235
Clomipramine	2.005	1.391	0.781	4.144	2.353	2.473	4.115	3.910
Desipramine	1.595	1.031	2.134	3.020	2.015	2.341	3.171	2.888
Fluphenazine	2.159	1.496	1.683	4.554	2.922	2.688	4.067	3.352
Imipramine	1.670	1.097	1.391	3.535	2.082	3.158	3.133	3.020
Perphenazine	2.283	1.393	1.635	4.305	2.997	3.092	3.256	3.070
Prochlorperazine	2.614	1.726	1.452	4.878	1.843	2.421	4.395	3.523
Promazine	1.890	1.165	1.556	3.492	2.338	2.808	3.794	3.294
Promethazine	1.833	1.508	1.693	4.081	1.132	3.169	3.069	3.216
Thioridazine	2.448	1.752	2.113	4.260	2.055	2.924	3.182	4.635
Trifluoperazine	2.388	1.820	1.778	4.948	1.792	2.644	5.022	3.632
Triflupromazine	1.976	1.514	1.960	4.409	2.533	2.638	3.790	4.117

**Table 3:** Biological activity/physicochemical properties values of selected antipsychotic drugs

Compound	−Δ*H* [kcal/mol]	log 1/D	log (1/ED_50_ × 10^−3^)	log ATPase activity
Aceperon	0.37	−1.623	—	—
Acetophenazine	—	—	2.12	—
Amitriptiline	—	—	1.85	—
Benperidol	1.82	1.523	—	—
Chlorpromazine	5.12	−0.301	2.52	−0.21
Chlorprothixene	0.19	0.022	2.57	—
Clomipramine	—	—	2.22	—
Desipramine	—	—	1.58	—
Dixyrazine	4.78	−0.398	—	—
Droperidol	0.96	1.284	—	—
Fluphenazine	0.00	1.097	—	—
Haloperidol	5.16	1.284	—	—
Imipramine	—	—	1.79	−0.99
Levomepromazine	3.42	−0.330	—	—
Nortriptiline	—	—	1.76	—
Perphenazine	—	—	2.63	0.09
Prochlorperazine	0.00	0.137	2.75	0.87
Promazine	—	—	2.05	−1.17
Promethazine	—	—	1.82	−0.37
Spiroperidol	0.69	2.000	—	—
Thioproperazine	0.00	0.602	—	—
Thioridazine	—	—	2.72	0.79
Trifluoperazine	—	—	2.92	1.69
Trifluopromazine	—	—	—	0.27
Triperidol	5.78	1.456	—	—

First of all, the analysis of PCA was performed for the geometry-optimized structures. At the outset, a PCA analysis was carried out of only non-empirical data in order to check whether and to what extent they could be useful in order to classify the compounds in question. The following results were obtained: PC1 is about 50.2%, PC2 is equal to 22.6% and PC3 ~10.9%. PC1 has the greatest impact on the maximum positive charge on atoms (MAX_POS), the difference between the highest positive and negative charge on atoms (Δ*Q*) and isotropic polarization (IPOL). It is interesting that electrons are considered polar parameters, while bulk parameters (TE, ESE) have an approximately smaller impact on PC1. On the other hand, PC2 influences mostly the largest negative electron charge on atoms (MAX_NEG) and the energy of the highest occupied molecular orbital (E_HOMO). PC3 has the greatest impact on IPOL and again Δ*Q* but also appears MAX_NEG and MAX_POS with smaller impact Fig. 1.

Factor analysis (FA) performed with the same calculation conditions coincides completely with the results for the molecules obtained by PCA method (FA1 ~50.2% and FA2 ~22.6%).

**Figure 2:**
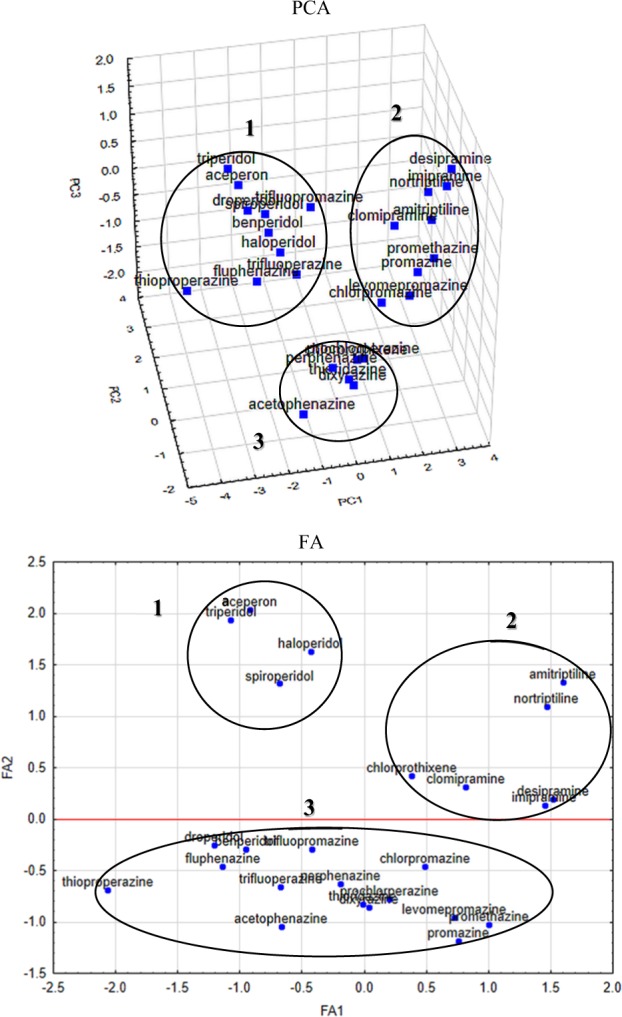
Three-dimensional scatter plots of the scores derived from the quantum-chemical calculations of structural parameters of the first three components obtained using PCA (PC1 50.21%, PC2 22.63%, PC3 10.89%) together with two-dimensional plot of scores the first two factors obtained by FA (FA1 50.21%, FA2 22.63%)

Figure 2 represents scatter plots of the scores according to performed PCA and FA. Analyzing PCA 3D scatter plots of the score we can distinguish three type of clusters; the first one includes: acepron, benperidol, droperidol, haloperidol, spiroperidol, triperidol, and also fluphenazine, trifluopromazine, trifluoperazine, thioproperazine; the second cluster containes: amitriptiline, clomipramine, desipramine, imipramine, nortriptiline also chlorpromazine, levomepromazine, promazine, promethazine; the third cluster includes: acetophenazine, chlorprothixene, dixyrazine, perphenazine, prochlorperazine, and thioridazine. We can observe that the first cluster mostly involves neuroleptics derived from butyrophenone and other very potent neuroleptics, the second one mostly belongs to tricyclic antidepressants (dibenzoazepine and dibenzoheptadiene derivatives), and the third one mostly to tricyclic neuroleptics.

On the other hand, analyzing FA 2D scatter plots of scores we can also distinguish three types of clusters: the first one includes: acepron, droperidol, haloperidol, and spiroperidol; the second: amitriptiline, clomipramine, desipramine, imipramine nortriptiline, and chlorprothixene and finally the third one includes all the others studied psychoactive drugs.

Statistically significant Gaussian parameters characterizing biological activity are presented in the following equations:

1 $${\mathrm{log}}\,\left( {1{\mathrm{/ED}}_{50} \times 10^{ - 3}} \right) = k_0 - 0.9150TE\left( { \pm 0.1165} \right)$$

*R* = 0.9150, *R*^2^ = 0,8372, *F* = 61.7126, *s* = 0.1813, *p* < 0.0001, *n* = 14, *k*_0_ = 0

2 $${\mathrm{log}}\,{\mathrm{ATPase}}\,{\mathrm{activity}} = k_0 - 0.7896{\mathrm{E}}\_{\mathrm{LUMO}}\left( { \pm 0.2319} \right)$$

*R* = 0.7896, *R*^2^ = 0.6235, *F* = 11.5949, *s* = 0.6559, *p* = 0.0114, *n* = 9, *k*_0_ = 0

Similarly, statistically significant Gaussian parameters characterizing chromatographic retention values are presented below:

3 $${\mathrm{log}}\,{\mathrm{k}}_{{\mathrm{AGP}}} = k_0 - 1.2633{\mathrm{TE}}\,\left( { \pm 0.1299} \right) - 0.4892{\mathrm{TDM}}\,\left( { \pm 0.1299} \right)$$

*R* = 0.9608, *R*^2^ = 0.9231, *F* = 60.0945, *s* = 0.3036, *p* < 0.0001, *n* = 13, *k*_0_ = 0

4 $${\mathrm{log}}\,{\mathrm{k}}_{{\mathrm{IAM}}} = k_0 - 0.7713{\mathrm{TE}}\,\left( { \pm 0.1919} \right)$$

*R* = 0.7713, *R*^2^ = 0.5949, *F* = 16.1514, *s* = 0.6648, *p* = 0.0020, *n* = 13, *k*_0_ = 0

5 $${\mathrm{log}}\,{\mathrm{k}}_{{\mathrm{w}}7.4{\mathrm{Su}}} = k_0 - 0.8364{\mathrm{TE}}\left( { \pm 0.1652} \right)$$

*R* = 0.8364, *R*^2^ = 0.6996, *F* = 25.6215, *s* = 0.5724, *p* = 0.0004, *n* = 13, *k*_0_ = 0

6 $${\mathrm{log}}\,{\mathrm{k}}_{\mathrm{w}}2.5{\mathrm{Sp}} = k_0 + 0.6378{\mathrm{ESE}}\,\left( { \pm 0.2322} \right)$$

*R* = 0.6378, *R*^2^ = 0.4068, *F* = 7.5449, *s* = 0.8044, *p* = 0.0190, *n* = 13, *k*_0_ = 0

7 $${\mathrm{log}}\,{\mathrm{k}}_{{\mathrm{w}}7.3{\mathrm{Al}}} = k_0 - 0.5936E\_LUMO\left( { \pm 0.2426} \right)$$

*R* = 0.5936, *R*^2^ = 0.3524, *F* = 5.9859, *s* = 0.8405, *p* = 0.0324, *n* = 13, *k*_*0*_ = 0

8 $${\mathrm{log}}\,{\mathrm{k}}_{{\mathrm{w}}11.7{\mathrm{Un}}} = k_0 - 0.6671{\mathrm{E}}\_{\mathrm{LUMO}}\,\left( { \pm 0.2246} \right)$$

*R* = 0.6671, *R*^2^ = 0.4450, *F* = 8.8226, *s* = 0.0127, *p* = 0.7780, *n* = 13, *k*_0_ = 0

Obtained results in most with TE and E_LUMO (TDM and ESE in individual cases) may indicate the specific nature of interactions between the drug molecule and the receptor.

In the final step, progressive stepwise multiple regression analysis was performed but with the use of a huge set of additional descriptors obtained by the professional software for the analyzed compounds with owned biological activity and chromatographic retention parameter values. Model equations for statistically significant descriptors are presented on below set of derived equations.

Biological activity/physicochemical properties values described by the Dragon software are as follows:

9 $$\begin{array}{l} - \Delta {\mathrm{H}}\,\left[ {{\mathrm{kcal/mol}}} \right] = k_0 + 0.5040{\mathrm{CATS}}2{\mathrm{D}}\_09\_{\mathrm{DA}}\\ \left( { \pm 0.1326} \right) - 1.0654{\mathrm{RDF}}095{\mathrm{v}}\,\left( { \pm 0.2195} \right) + \\ 0.6750\,{\mathrm{H}}4{\mathrm{m}}\,\left( { \pm 0.2357} \right)\end{array}$$

*R* = 0.9537, *R*^2^ = 0.9095, *F* = 30.1882, *s* = 0.3472, *p* < 0.0001, *n* = 13, *k*_0_ = 0

10 $$\begin{array}{l}{\mathrm{log}}\,1/{\mathrm{D}} = k_0 + 0.5926\,{\mathrm{H}} - 053\left( { \pm 0.0630} \right) + 0.4112{\mathrm{PJI}}3\\ \left( { \pm 0.0614} \right) + 0.3169{\mathrm{AVS}}\_{\mathrm{B}}\left( {\mathrm{e}} \right)\left( { \pm 0.0584} \right)\end{array}$$

*R* = 0.9858, *R*^2^ = 0.9718, *F* = 103.6188, *s* = 0.1937, *p* < 0.0001, *n* = 13, *k*_0_ = 0

11 $$\begin{array}{l}{\mathrm{log}}\,\left( {1{\mathrm{/ED}}_{50} \times 10^{ - 3}} \right) = k_0 + 0.8553{\mathrm{DISPm}}\,\left( { \pm 0.0647} \right) + \\ 0.3122{\mathrm{Mor}}08{\mathrm{u}}\,\left( { \pm 0.0585} \right) + 0.1797{\mathrm{MATS}}6{\mathrm{s}}\,\left( { \pm 0.0566} \right)\end{array}$$

*R* = 0.9873, *R*^2^ = 0.9748, *F* = 128.4855, *s* = 0.1813, *p* < 0.0001, *n* = 14, *k*_0_ = 0

12 $$\begin{array}{l}{\mathrm{log}}\,{\mathrm{ATPase}}\,{\mathrm{activity}} = k_0 + 0.7842{\mathrm{Eig}}04\_{\mathrm{AEA}}\\ \left( {{\mathrm{bo}}} \right)\,\left( { \pm 0.0747} \right) - 0.2958{\mathrm{GATS}}5{\mathrm{s}}\,\left( { \pm 0.0747} \right)\end{array}$$

*R* = 0.9891, *R*^2^ = 0.9783, *F* = 135.5797, *s* = 0.1699, *p* < 0.0001, *n* = 9, *k*_0_ = 0

Analogous chromatographic retention parameters described by the Dragon software are presented below:

13 $$\begin{array}{l}{\mathrm{log}}\,{\mathrm{k}}_{{\mathrm{AGP}}} = k_0 + 1.2632{\mathrm{H}}1{\mathrm{v}}\,\left( { \pm 0.1175} \right) - \\ 0.2847{\mathrm{DISPi}}\,\left( { \pm 0.0994} \right) - 0.1993H2u\,\left( { \pm 0.0859} \right)\end{array}$$

*R* = 0.9804, *R*^2^ = 0.9612, *F* = 74.4953, *s* = 0.2272, *p* < 0.0001, *n* = 13, *k*_0_ = 0

14 $$\begin{array}{l}{\mathrm{log}}\,{\mathrm{k}}_{{\mathrm{IAM}}} = k_0 - 0.8764{\mathrm{X}}1{\mathrm{A}}\,\left( { \pm 0.0826} \right) - 0.4126{\mathrm{GATS}}5{\mathrm{m}}\\ \left( { \pm 0.0697} \right) - 0.2502{\mathrm{Mor}}06{\mathrm{p}}\,\left( { \pm 0.0817} \right)\end{array}$$

*R* = 0.9845, *R*^2^ = 0.9692, *F* = 94.6584, *s* = 0.2024, *p* < 0.0001, *n* = 13, *k*_0_ = 0

15 $$\begin{array}{l}{\mathrm{log}}\,{\mathrm{k}}_{{\mathrm{w}}7.4Su} = k_0 + 0.7018{\mathrm{R}}2{\mathrm{v}}\,\left( { \pm 0.1133} \right) - \\ 0.3803{\mathrm{CMC}} - 50\,\left( { \pm 0.1133} \right)\end{array}$$

*R* = 0.9506, *R*^2^ = 0.9036, *F* = 46.8890, *s* = 0.3400, *p* < 0.0001, *n* = 13, *k*_0_ = 0

16 $$\begin{array}{l}{\mathrm{log}}\,{\mathrm{k}}_{{\mathrm{w}}2.5{\mathrm{Sp}}} = k_0 + 0.6116\,{\mathrm{R}}1{\mathrm{e}} + \left( { \pm 0.1063} \right) - 0.3475{\mathrm{Mor}}09{\mathrm{i}}\\ \left( { \pm 0.1088} \right) + 0.3378\,{\mathrm{B}}08\left[ {{\mathrm{C}} - {\mathrm{N}}} \right]\,\left( { \pm 0.1107} \right)\end{array}$$

*R* = 0.9532, *R*^2^ = 0.9086, *F* = 29.8380, *s* = 0.3490, *p* < 0.0001, *n* = 13, *k*_0_ = 0

17 $$\begin{array}{l}{\mathrm{log}}\,{\mathrm{k}}_{{\mathrm{w}}7.3{\mathrm{Al}}} = k_0 + 1.2948\,{\mathrm{H}}6{\mathrm{m}}\,\left( { \pm 0.1279} \right) - 1.4089\,{\mathrm{ATS}}6{\mathrm{s}}\\ \left( { \pm 0.1882} \right) + 0.8301{\mathrm{H}}4{\mathrm{i}}\,\left( { \pm 0.1638} \right)\end{array}$$

*R* = 0.9718, *R*^2^ = 0.9444, *F* = 50.8709, *s* = 0.2725, *p* < 0.0001, *n* = 13, *k*_0_ = 0

18 $$\begin{array}{l}{\mathrm{log}}\,{\mathrm{k}}_{{\mathrm{w}}11.7{\mathrm{Un}}} = k_0 + 0.4900{\mathrm{ALOGP}}2\,\left( { \pm 0.1072} \right) + \\ 0.3580{\mathrm{HATS}}1\left( { \pm 0.0598} \right) + 0.3380{\mathrm{R}}8{\mathrm{v}}\,\left( { \pm 0.1004} \right)\end{array}$$

*R* = 0.9873, *R*^2^ = 0.9748, *F* = 115.9598, *s* = 0.1834, *p* < 0.0001, *n* = 13, *k*_0_ = 0

The full list of molecular descriptors obtained from Dragon software with their designations is presented in Table 4.

**Table 4:** List of statistically significant molecular descriptors characterizing biological activity/physicochemical properties/chromatographic retention obtained from Dragon software (Eqs. 9–18)

Descriptor	Full name	Block
Biological activity/physicochemical properties		
CATS2D_09_DA	CATS (Chemically Advanced Template Search) 2D Donor–Acceptor at lag 09	CATS2D
RDF095v	Radial Distribution Function - 095 / weighted by van der Waals volume	RDF descriptors
H4m	H autocorrelation of lag 4/weighted by mass	GETAWAY (GEometry, Topology, and Atom-Weights AssemblY) descriptors
H-053	H attached to C0(sp3) with 2X attached to next C	Atom-centered fragments
PJI3	3D Petitjean shape index	Geometrical descriptors
AVS_B(e)	Average vertex sum from Burden matrix weighted by Sanderson electronegativity	2D matrix-based descriptors
DISPm	Displacement value/weighted by mass	Geometrical descriptors
Mor08u	Signal 08/unweighted	3D-MoRSE (3D-Molecule Representation of Structures based on Electron diffraction) descriptors
MATS6s	Moran autocorrelation of lag 6 weighted by I-state	2D autocorrelations
Eig04_AEA(bo)	eigenvalue n. 4 from augmented edge adjacency mat. weighted by bond order	Edge adjacency indices
GATS5s	Geary autocorrelation of lag 5 weighted by I-state	2D autocorrelations
Retention parameters		
H1v	H autocorrelation of lag 1/weighted by van der Waals volume	GETAWAY descriptors
DISPi	Displacement value/weighted by ionization potential	Geometrical descriptors
H2u	H autocorrelation of lag 2/unweighted	GETAWAY descriptors
X1A	Average connectivity index of order 1	Connectivity indices
GATS5m	Geary autocorrelation of lag 5 weighted by mass	2D autocorrelations
Mor06p	Signal 06/weighted by polarizability	3D-MoRSE descriptors
R2v	R autocorrelation of lag 2/weighted by van der Waals volume	GETAWAY descriptors
CMC-50	Ghose–Viswanadhan–Wendoloski CMC drug-like index at 50%	Drug-like indices
R1e+	R maximal autocorrelation of lag 1/weighted by Sanderson electronegativity	GETAWAY descriptors
Mor09i	Signal 09/weighted by ionization potentia	3D-MoRSE descriptors
B08[C-N]	Presence/absence of C-N at topological distance 8	2D Atom Pairs
H6m	H autocorrelation of lag 6/weighted by mass	GETAWAY descriptors
ATS6s	Broto–Moreau autocorrelation of lag 6 (log function) weighted by I-state	2D autocorrelations
H4i	H autocorrelation of lag 4/weighted by ionization potential	GETAWAY descriptors
ALOGP2	Squared Ghose–Crippen octanol-water partition coeff. (logP^2)	Molecular properties
HATS1v	Leverage-weighted autocorrelation of lag 1/weighted by van der Waals volume	GETAWAY descriptors
R8v	R autocorrelation of lag 8/weighted by van der Waals volume	GETAWAY descriptors

Performed predictions with the use of professional software and the wide range of molecular descriptors provide more detail information about the studied molecules.

The obtained statistically significant molecular descriptors belong to different classes but we can distinguish some of the common one’s classes between them. In our opinion it is difficult to indicate the most important equation between the proposed because the datasets are relatively small, therefore we wanted to focus rather on the same block of descriptors appearing in the presented equations.

In the case of biological activity/physicochemical properties of analyzed psychoactive drugs, the most often appeared geometrical descriptors together with GETAWAY and atom fragments descriptors followed by the 2D autocorrelations and 3D-MoRSE descriptors. Experimental equations confirmed the very important role of geometric and topologic properties of the molecules together with electronic ones. Furthermore, interesting information we received also analyzing the retention parameters values on different chromatographic columns and in different chromatographic conditions. There was a confirmation in eight of the experimental cases particularly important role of GETAWAY descriptors and also molecular properties descriptors (e.g. ALOGP2, characterizing octanol-water partition coefficient) followed by appeared again 2D autocorrelations and 3D-MoRSE descriptors, so we can observe that similar group of descriptors play a dominant role in pharmacological role and physicochemical properties of examined psychoactive structures.

## Conclusion

Based on the above discussion, the results can be drawn as follows. The largest influence on the values of both biological activity/physicochemical properties and chromatographic retention parameters among the 10 quantum-chemical parameters considered (Gaussian software) are most often the total energy (TE) and frontier orbital energy LUMO (E_LUMO). On the other hand, we can distinguish GETAWAY (GEometry, Topology, and Atom-Weights AssemblY) descriptors next to 3D-MoRSE (3D-Molecule Representation of Structures based on Electron diffraction) descriptors from Dragon software, which leads to the assumption that a functional dependency exists. These parameters seem to be particularly important for psychoactive activity and properties of analyzed structures, which is related to the hypotheses regarding the mechanism of action of compounds with this type of elements of structure and mostly their pharmacological classification. It seems advisable also to continue research in the extended database of molecules and their experimental values of pharmacological/physicochemical properties.
